# Clinical Society of Maryland

**Published:** 1896-04

**Authors:** H. O. Reik

**Affiliations:** Secretary; 525 North Howard Street; Baltimore


### SOCIETY REPORTS.

### CLINICAL SOCIETY OF MARYLAND.

BALTIMORE, February 7, 1896.

The meeting was called to order by the president, DR. J. M. HUNDLEY.

DR. J. WHITRIDGE WILLIAMS read a paper on the "Induction of Premature Labor for other than the Usual Indications," of which the following is an abstract:

The most usual indications for the induction of premature labor are either moderate degrees of pelvic contraction or progressive renal insufficiency. During the past year it has been my lot to meet with two cases which demanded the operation for other than the usual indications; the first being a case of acute dilatation of the heart following mitral insufficiency, and the second a case of acute pyelitis which directly threatened the life of the mother.

CASE 1. Mrs. M. V., aged thirty-four, married eight years, multipara. Up to three years ago personal and family history negative, but at that time she was confined to bed with acute rheumatism, the knees and ankles being the parts affected. Present pregnancy perfectly normal up to the latter part of November, 1894, when she began to suffer from dyspnea. On Christmas day she applied for aid at the Johns Hopkins Hospital, obstetrical department, and my assistant saw her. She was suffering from broken heart-compensation, and presented a somewhat dilated heart and a distinct mitral regurgitant murmur. She was placed upon digitalis, and later upon iodide of potash and Blaud's pills, and was comfortable up to January 10, 1895, when my assistant was again called and found her in a serious condition. I was at once sent for and found her sitting up in bed fairly fighting for air. She was intensely cyanotic, with a pulse from 150 to 160 per minute. The heart was markedly dilated, laboring intensely, and murmurs were heard at all the valves. Lungs edematous. Hypodermics of digitalis were given, as well as a considerable quantity of whisky, but in spite of this the dyspnea and pulmonary edema increased. I then tried the effects of blood-letting, removing about a pint from the veins of the right arm, this giving temporary relief. I followed it by a second venesection, at the conclusion of which she was able to lie down and sleep. During the following day her condition again became so serious that we determined to induce premature labor. Under the strictest antiseptic precautions a silk-covered bougie was passed into the uterus. During the next three days several other and larger bougies were introduced, and eventually she gave birth to a large child perfectly spontaneously. Immediately after delivery her condition improved, but the puerperium was not entirely normal. Her rise of temperature seemed to be due to a lighting up of the old endocarditis rather than to sepsis. The heart recovered its compensation, and she was able to leave the hospital on the twelfth day.

CASE 2. Mrs. S., aged thirty, primipara; family and personal history negative. I was first consulted when she was about four months pregnant, suffering with a great deal of pain in the lower part of the abdomen and obliged to pass small quantities of urine almost constantly. From the history I suspected a retroflexed pregnant uterus, but upon examination found that organ in its normal position. The urine was examined, had a specific gravity of 1016, acid reaction, and under the microscope showed large numbers of leucocytes. Diagnosis of acute cystitis was made, the patient placed under treatment, and in two weeks pus had disappeared from the urine. All went well with her until the latter part of August, when she was suddenly seized with intense pains in the left renal region, accompanied by chills and fever. She was then in Chicago, and recovered sufficiently under medical treatment to return to her family in Virginia. For several weeks she did very well, but was again seized with a similar attack which did not promptly yield to treatment, and she was brought to Baltimore. She recovered from this attack, which was in September, but had a recurrence in October, when her condition was more serious. She emaciated rapidly, was constantly nauseated, and the urine was loaded with leucocytes and both granular and hyaline casts. After consultation it was deemed best to empty the uterus. Chloroform was given, but before she had become completely anesthetized her respiration ceased, and it was with the greatest difficulty that she could be resuscitated. Bougies were then introduced without anesthesia, but as the contractions were so feeble, I had to dilate the cervix manually and extract the child. This operation was done under ether. The child died thirty-six hours after birth, apparently from a heart lesion. The mother improved rapidly, and when last heard from was in excellent condition.

DISCUSSION.

DR. L. E. NEALE: It seems to me that the method of dilatation by bougies is rather slow for such cases, and if possible that the condition of the patient would permit, the result might have been brought about in an easier and more rapid way. In the case which I saw with Dr. Williams, that had to be resorted to eventually after thirty-six hours' delay with the bougie. I remember saying that it was best that woman should be delivered as soon as possible. I have had some small experience with the slowness of this instrument's working, and have at times been forced to abandon the bougie, and placing the patient under an anesthetic, to dilate and deliver. In the use of glycerine, which has been advised, I believe the best authorities are now strongly opposed to such a thing. My own results have not been satisfactory. For my part, if the case called urgently for delivery I would, as soon as her condition demanded it, not delay for bougies, but dilate and deliver as fast as possible. I think much valuable time may thus be saved.

Dr. Williams did not mention one little point which I have always considered of great importance—that is, the repeated introduction of the bougie in different directions and twisting it about between the membranes before letting it remain. I don't think the dangers of rupture are very serious. All such measures, though, are very slow and liable to fail, and there is no way of foretelling in which case they will succeed and in which fail. Where there is no cause for haste such measures would be applicable, but where such cause does exist more rapid measures should be employed.

DR. C. H. JONES: I arrived too late to hear Dr. Williams's paper,

I am sorry to say, for I have a case to report and upon which I would like the opinion of the members. It is that of a young woman twenty-four years of age, married, and pregnant for the first time. She has a marked double aortic murmur without any history of rheumatism, scarlet fever, or any of the diseases that would produce the heart trouble. She is in good health, and did not know she had a heart until a life insurance agent examined her and told her she might drop dead at any minute. What I want to know is this: Whether it is advisable to wait for term, or to perform an abortion, or induce premature labor in the case. She is now pregnant nearly five months.

DR. J. W. WILLIAMS: I would say in regard to this case, that I think any interference would be unjustifiable. The less you do for her the better, unless she develops some other symptoms. I have seen several cases of marked heart lesions go through labor without any trouble, and the heart cases, too, bear an anesthetic very well. There is no need to induce labor unless some emergency arises. In my first case we induced premature labor in order to take the strain off the heart. I should advise you to wait till the end of the pregnancy.

DR. L. E. NEALE: I can report a case which occurred one month ago, where the patient had both aortic and mitral lesions when she came in the hospital. Nothing was done for her, and she had a perfectly normal labor. I can recall a case, on the other hand, where there was no valvular murmur heard, but there was a very slow pulse, and which proved fatal. It was a criminal abortion, and she was taken sick on the train. She had several fainting spells. Labor was perfectly normal, but immediately afterwards she went into collapse state and died. No post mortem was made, so I can say nothing about the condition of the heart, but I suspect there was trouble with the muscular structure rather than with the valves. In Dr. Jones's case I should do nothing.

DR. W. H. WELCH: I am not an obstetrician, but I have recently looked up the literature on the subject of heart trouble, and I noticed there the statement that patients pass through pregnancy and labor without any interference with the action of the heart in these cases. It is not clear why compensation should be broken in pregnancy. As regards the relative prognosis in aortic or mitral disease, I think there is no question that the patient is more comfortable with aortic than with mitral trouble. A patient with aortic insufficiency may live out a long life of hard work, and the question has been raised whether they should be refused life insurance. When compensation is broken, however, they run a more rapid and fatal course than those of mitral insufficiency.

DR. J. FRANK CROUCH: Speaking of broken compensation, I would like to report a case that came under my notice. The patient was a young woman of twenty-four, pregnant for the first time. She was under the charge of one of my friends, and during his absence from the city I was called to see her. I found a well marked mitral regurgitation and considerable edema. Placed her upon digitalis, and she went to the end of pregnancy without any disturbance of the heart at all. She went through a normal first stage of labor, but when the head came into the pelvis she sudddenly became cyanotic and presented some alarming symptoms. I was sent for to assist. Pulse was 125, and she was in a grave condition. The question of anesthetics was raised, and we decided to give it. Chloroform was used, and the heart seemed to grow stronger. The child was delivered by traction. Compensation was established and inside of twenty-four hours she was comfortable, but four months later she dropped dead. I have seen other cases of broken compensation occurring during labor. Fifteen months ago I was called to see a woman who was gasping for breath when I reached her bedside. She rallied under digitalis and went on comfortably for two weeks, when she had another attack of dilatation. Labor was induced. She was delivered, and since that time has had no trouble. I take it that in cases of well compensated valvular trouble it is unwise to interfere until compensation is broken.

DR. RANDOLPH recited a case of recovery from "Sympathetic Ophthalmia," with exhibition of patient.

By way of preface, I should say that there was no objective features about this case which would strike the general practitioner, but inasmuch as recoveries from this disease are exceedingly rare, and as this is the first case of the kind which has been brought before the society, I thought it would be interesting simply to see one who has had such an experience. You all know that sympathetic ophthalmia is traceable to a penetrating wound of the eyeball, that is to say, an eye is injured, irido-cyclitis follows, and in a certain length of time, ordinarily from three to ten weeks, inflammation breaks out in the other eye, and this inflammation is called sympathetic ophthalmia. Sympathetic ophthalmia may be defined then as a plastic inflammation of the iris, ciliary body, and choroid of one eye, having its origin in a traumatic inflammation of the same parts in the other eye. Injuries of this character as a rule destroy sight at once, or certainly in a very few days after the injury. Eyes then which have been rendered sightless by penetrating wounds are regarded as dangerous eyes and liable to give rise to sympathetic trouble, and constantly they are removed. Such a course requires little deliberation on the part of the surgeon, but there is another class of cases seldom seen where vision is preserved in the injured eye after the outbreak of the sympathetic inflammation, and the case which I exhibit here to-night belongs to this variety. Such a case calls for no little deliberation in its treatment. This man came to the dispensary of the Johns Hopkins Hospital early last May. I found a penetrating wound of the upper and inner border of the corneo-scleral region, and at the bottom of the anterior chamber there was a faint line of what appeared to be pus. The vision was reduced to the ability to count fingers at six feet. He was given the usual treatment in such cases—one per cent. solution of atropine to be used every three hours, and a compress bandage. He got worse, however, and returned in three days with the anterior chamber nearly full of pus and only slight perception left. I performed paracentesis of the anterior chamber, and fortunately there was no reformation of the pus. The condition went on to get better, and in the fifth week after the accident he could count fingers at fifteen feet. He disappeared from the clinic, and came back in four days with the complaint that he could scarcely see anything out of the right eye. An examination revealed a typical case of sympathetic uveitis. The media was so cloudy at this time that it was impossible to get a satisfactory view of the fundus. The vision of this eye was $\frac{16}{200}$, while that of the injured eye, the eye which had caused all the trouble, was still the ability to count fingers at fifteen feet. If the left eye had been blind I would have removed it, but the fact that good vision was still present in it and the possibility of its becoming ultimately the better eye, contra-indicated any radical measure. There is a case on record where a man in just such a plight as this one lost the sympathetically affected eye, and the exciting or injured eye became the useful eye. There was no change in my patient until the end of the ninth week, when the injured eye became suddenly very soft and in the course of a few days light perception disappeared. There was then no longer any necessity of keeping this eye, and thinking that as the focus of the trouble it might still be sending pernicious impulses to the other eye, it was removed. Not long after this the sight in the other eye begun to improve, and now, nine months after the development of the sympathetic affection, he has $\frac{16}{40}$ vision, and can read some of the letters on the next lower line. The prognosis in these cases must be given with a certain amount of reserve, for lapses are common. As a rule, however, a patient who goes a year without relapse presents conditions which justify a favorable prognosis. In the past twenty years there have been reported about eighteen cases of recovery from sympathetic ophthalmia. This case is interesting then, not only because it is one of recovery from sympathetic ophthalmia, but because vision was preserved in the injured eye a considerable length of time after the outbreak of the sympathetic affection.

DISCUSSION.

DR. E. J. BERNSTEIN: I was called recently by Dr. Hall to see a colored man who had received an injury to his eye in June last. He had gone from bad to worse until he had only light perception, and in the other eye had symptoms of sympathetic ophthalmia—I mean signs of papillitis—present. I removed the offending eye and his vision in the other improved. It was done only three weeks ago, though.

DR. W. H. WELCH: What were the anatomical lesions in this case? I believe ophthalmologists make a distinction between sympathetic irritation without anatomical lesions and sympathetic inflammation of the eye with changes in its structure. To what extent, then, were the symptoms in this case due to irritation and what to inflammation, and how far had the lesions retrogressed?

DR. RANDOLPH: Cases such as the one reported by Dr. Bernstein have been observed by Noyes, Milles, Frost, and others, but they should not be regarded as examples of true sympathetic ophthalmia. As I have remarked, true sympathetic ophthalmia is a plastic uveitis; in other words, an inflammation of the iris, choroid, and ciliary body, an inflammation accompanied with the usual symptoms of a plastic irido-cyclitis, with which you are familiar. Dr. Bernstein's case should be called sympathetic papillitis, which, like sympathetic irritation, is a benign form of sympathetic ophthalmia. In both these varieties of the disease the sympathetic manifestation, as a rule, disappears entirely with the enucleation of the injured eye, while in true sympathetic ophthalmia, such as the one I have reported, the removal of the injured eye is followed by no improvement of the sympathetic eye. The improvement which was commenced in my case soon after the enucleation of the injured eye was probably a coincidence. In Dr. Bernstein's case the patient will recover normal vision, while in my case such a termination is impossible. In answer to Dr. Welch's question I may say that the anatomical lesions in the sympathetically affected eye consisted in two posterior synechiæ at the lower and outer edge of the pupil and opacities in the vitreous body, and now that the media are clear enough to permit a view of the eye-ground, there may be seen widespread changes in the choroid, consisting in pigment deposits, chiefly in the region of the papilla, and numerous whitish areas of degeneration.

H. O. REIK, M.D., Secretary.

525 *North Howard Street.*

